# The effect of dobutamine on ocular blood flow of healthy adults: A 3D pseudocontinuous aterial spin labelling study

**DOI:** 10.3389/fphys.2022.1003915

**Published:** 2022-11-29

**Authors:** Linkun Cai, Haijun Niu, Pengling Ren, Yawen Liu, Tingting Zhang, Dong Liu, Erwei Zhao, Liang Zhu, Jing Li, Penggang Qiao, Wei Zheng, Zhenchang Wang

**Affiliations:** ^1^ School of Biological Science and Medical Engineering, Beihang University, Beijing, China; ^2^ Department of Radiology, Beijing Friendship Hospital, Capital Medical University, Beijing, China; ^3^ Department of Ultrasound, Beijing Friendship Hospital, Capital Medical University, Beijing, China; ^4^ National Space Science Center, Chinese Academy of Sciences, Beijing, China; ^5^ National Research Center for Rehabilitation Technical Aids, Beijing, China

**Keywords:** dobutamine, 3D-pc ASL, ocular blood flow, risk factors, healthy adult

## Abstract

**Purpose:** Ocular blood flow (OBF) is an important risk factor for incidence, prevalence and progression of some ocular disorders. To date, there are very limited therapeutic options to increase OBF. This study investigated the effect of dobutamine on OBF of heathy adults using 3D pseudocontinuous arterial spin labelling (3D-pcASL), and explored the risk factors associated with OBF.

**Methods:** Forty-three healthy participants (86 eyes) were given an intravenous injection of dobutamine. We measured OBF using 3D-pcASL with a 3.0T- MRI scanner, OBF values were independently obtained by two doctors from the OBF map. We also collected physiological parameters using a vital signs monitor. The OBF and physiological parameters in the in the period before and after dobutamine injection states were obtained.

**Results:** OBF increased significantly after dobutamine injection using paired *t* test method (from 22.43 ± 9.87 to 47.73 ± 14.02 ml/min/100g, *p* < 0.001). Age, heart rate and systolic blood pressure were the main risk factors affecting OBF using logistic regression analysis (all *p* values < 0.05).

**Conclusion:** To the best of our knowledge, this is the first study observing the effect of dobutamine on OBF. Our findings indicated that intravenously injected dobutamine increased OBF, making it a possible option to counteract ocular vascular ischaemia in the future.

## Introduction

Ocular blood flow (OBF) is an important risk factor for incidence, prevalence and progression of some ocular disorders such as glaucoma (Nakazawa 2016), diabetic retinopathy (Ueno et al., 2021), and ocular ischaemic syndrome (Lee et al., 2022). To date, there are very limited therapeutic options to increase OBF. Several peripheral vasodilators have been proposed as possible options to increase OBF and counteract ocular vascular ischaemia. For example, niacin therapy (oral dose of 500 mg) has been shown to increase choroidal blood volume by 39% after 30 min (Rahimy et al., 2016). Sildenafil, a phosphodiesterase five inhibitor, has been proposed to increase OBF. One hour after administration of the drug, choroidal blood flow showed increased peak systolic velocity, end-diastolic velocity and mean velocity values in the ophthalmic and short posterior ciliary arteries (Harris et al., 2008; Koksal et al., 2005). Intravenous administration of 150 mg moxaverine increased choroidal blood flow in healthy subjects by smooth muscle relaxation and subsequent peripheral vasodilation and improved blood rheology (Berg, Andersson, and Ryden 1988; Pemp et al., 2012), but oral administration of moxaverine did not increase OBF (Schmidl et al., 2013). Treatment with Ginkgo biloba extract for normal-tension glaucoma resulted in a significant increase in mean retinal capillary flow and velocity compared to placebo (Harris et al., 2018). Cannabinoid receptors are present in and around the blood vessels of the eye, so oral intake of 5 mg dronabinol induced a significant increase in total retinal blood flow (Hommer et al., 2021). However, these drugs usually have certain side effects, including flushing, headache, nasal congestion, gastric discomfort and allergic reactions (Moreira et al., 2000; Hoff Roland and Nergard, 2012). Therefore, there is no standard treatment protocol to increase OBF.

Dobutamine is a sympathomimetic amine that primarily stimulates myocardial β-1 adrenergic receptors, increases cardiac contractility without causing vasoconstriction or tachycardia, and is often prescribed as a contractile agent for congestive heart failure (Ruffolo 1987). Clinically, dobutamine increases cardiac output by selectively increasing the volume per beat, which can be used to assess the effect of increased cardiac work on impaired coronary perfusion (Pousset et al., 1995). The systemic hemodynamic effects of dobutamine have been well described (Akkermans et al., 2020; Szarpak et al., 2022) but there has been no research discussing or exploring the effect of dobutamine on OBF.

Ocular imaging techniques have advanced dramatically in recent decade, including fundus fluorescence angiography (FFA), indocyanine green angiography (ICGA), and optical coherence chromatography angiography (OCTA), provide insight into the vascular physiology of the eye. FFA and ICGA are invasive, require exogenous contrast agents, and are not suitable for people with contrast allergies. Optical techniques are influenced by the clarity of the ocular media and are sensitive to motion artefacts, and OCTA is qualitative and cannot quantify OBF (Grudzińska and Modrzejewska 2018). Therefore, a reliable measurement method for OBF remains to be found.

Arterial spin labelling (ASL) perfusion magnetic resonance imaging (MRI) is a noninvasive imaging modality whose main advantage is that blood itself acts as a tracer to quantify tissue microvascular perfusion. This technique has been widely used in clinical practice. One study evaluated chorioretinal blood perfusion in 20 young healthy adults and showed that ASL had high intra-day and inter-day reproducibility (Khanal et al., 2019). ASL has the potential to provide unique retinal perfusion information with good depth resolution as a complement to existing optical imaging techniques (Peng et al., 2011). Compared with other ASL imaging techniques, the 3D-pcASL technique has a higher labelling efficiency and intrasubject signal-to-noise ratio (Alsop et al., 2015), so it is currently recommended for use in clinical studies and has become an increasingly popular tool for researchers (Wang et al., 2021).

Within this context, we designed the present study to investigate the effect of the intravenous peripheral vasodilator dobutamine on OBF of heathy adults using 3D-pcASL method. We hypothesized that dobutamine is an effective way to increase OBF.

## Materials and methods

### Participants

The exclusion criteria were as follows: 1) coronary artery disease, diabetes mellitus, systemic hypertension and systemic diseases, which are considered to affect OBF; 2) ocular vascular diseases such as retinal vein occlusion; 3) typical contraindications to MRI; and 4) contraindications to dobutamine administration. All patients were instructed to refrain from cigarettes, tea, coffee, β-blockers, and antianginal medication for at least 24 h before the MR study. Among the 50 participants were initially enrolled in this project and seven participants were excluded based on exclusion criteria. Therefore, 43 healthy participants (86 eyes) were enrolled.

The intravenous dobutamine was well tolerated by the participants, as no adverse events occurred during the study period.

### Experimental design

The experimental design process is as follows: first, ASL data were collected before dobutamine injection. After a 10-min break, dobutamine was injected, and the ASL data after dobutamine injection was collected. Finally, dobutamine injection was stopped, and participants were allowed to leave when their HR and BP returned to the period before dobutamine injection. The acquisition time for each ASL data point was approximately 6 min.

During the periods before and after dobutamine injection, physiological parameters such as heart rate (HR) and blood pressure (BP) were monitored synchronously and continuously by a portable multi-parameter vital signs monitor (Mindray, BeneVision, Shenzhen, China). If a participant developed signs or symptoms of intolerance, the procedure was terminated.

### MRI data acquisition

All participants were examined using a 3.0T MRI scanner (Ingenia 3.0T; Philips Healthcare, Best, Netherlands) with a commercial body coil for transmission and a 16-channel head coil for reception. Conventional T2-weighted fast-spin-echo and 3D time-of-flight MR angiography images were obtained before the ASL sequence to exclude patients with obvious brain lesions or arterial stenosis.

Perfusion data were acquired by a 3D-pcASL sequence with a multislice fast spin-echo acquisition and background suppression. The gradient in 3D-pcASL was set to unbalanced scheme and the labeling parameters were optimized to decrease sensitivity to the off-resonance effect with: labeling duration = 1500msec and post labelling delay (PLD) = 1500msec. These parameters have been previously implemented to successfully image blood flow in the human eye (Khanal, Philip R.K. Turnbull et al. (2019). Images were acquired using the multishot TSE sequence with the following parameters: repetition time [TR] = 3903msec, echo time [TE] = 12msec, bandwidth [BW] = 7763.2Hz, flip angle (FA) = 90 ° , label distance = 90 mm, slice thickness = 3.5 mm, number of slices = 20, field of view (FOV) = 240 mm × 180 mm, in-plane resolution = 3 mm × 3 mm, matrix = 64 × 47, reconstruction matrix = 80, and number of excitations (NEX) = 2.

All participants were asked to keep their eyes closed, not to move their eyes, not to think of anything in particular, not to fall asleep during the examination. All participants were provided with foam pads and earplugs to reduce noise and head movements.

### ASL data quantification

**FIGURE 1:**
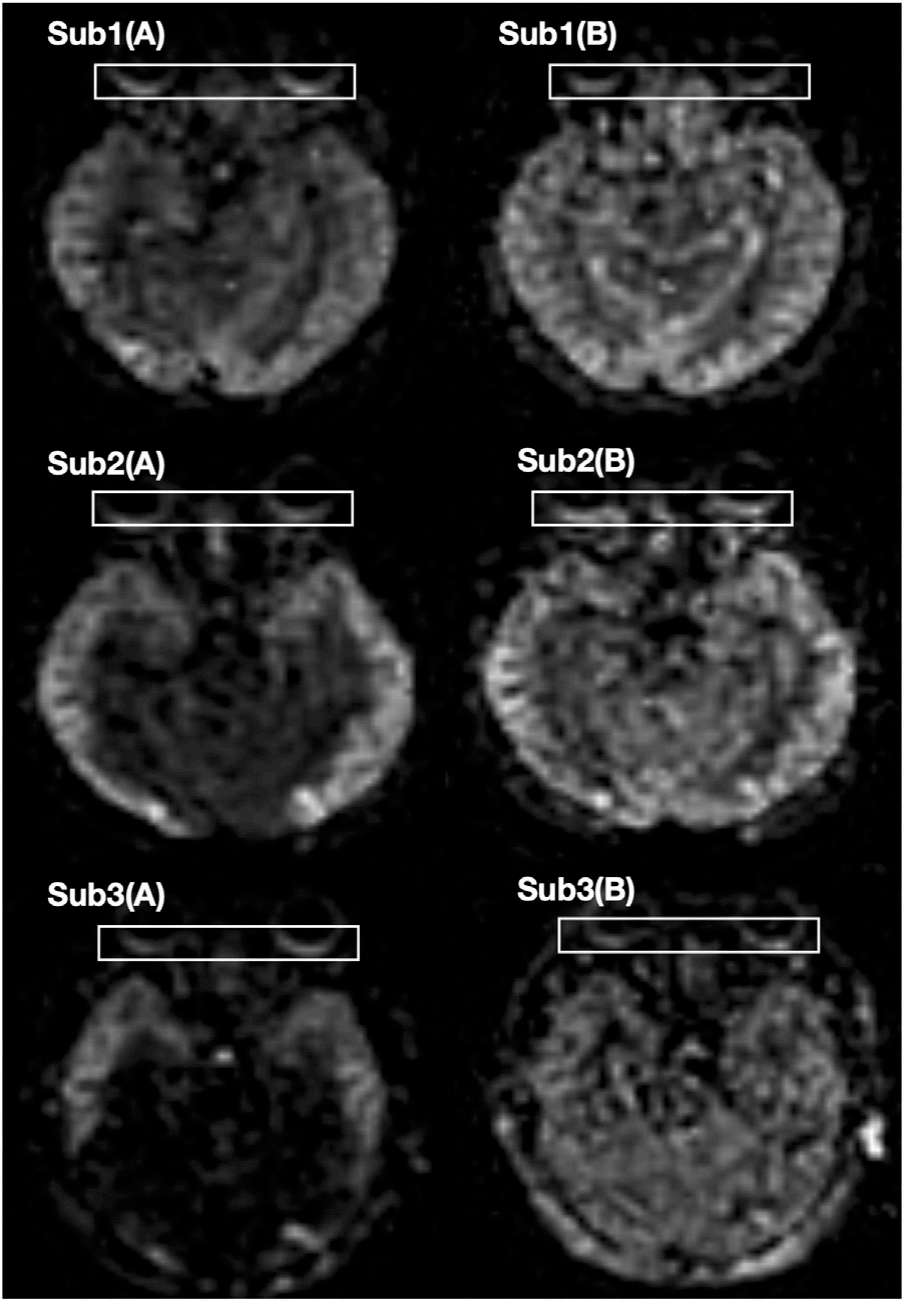
The OBF maps of three participants. **(A)** Before dobutamine injection **(B)** After dobutamine injection.

**FIGURE 2:**
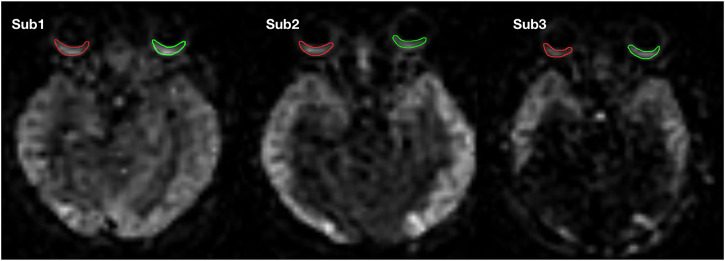
ROIs of one subject on the OBF map. Green represents the ROI of the left eye, and red represents the ROI of the right eye.

The OBF map was automatically derived from the 3D-pcASL images using a dedicated workstation (IntelliSpace Portal Release v. 7.0.4.20175, Philips) (Figure 1). A region of interest (ROI) was annotated at the level of the optic nerve in the fundus of the OBF map after discussion by a radiologist with 6 years of experience and an ophthalmologist with 4 years of experience (Figure 2). In the analyses using Kappa, the OBF values of the two annotators were consistent (Kappa value = 0.78).

### Statistical analysis

All statistical analyses were performed with IBM SPSS Statistics for Mac 19.0.0 (SPSS Inc., Chicago, Illinois, United States). The Shapiro–Wilk test was used to determine the distribution of the data and the paired *t* test was applied to detect significant differences between different states. Logistic regression models were built for physiological factors (independent variables), including age, myopic degree, BMI, and hemodynamic parameters (HR, systolic blood pressure and diastolic blood pressure) associated with OBF change (dependent variable). If the OBF change was greater than the average value, it was a positive sample; otherwise, it was a negative sample. *p* < 0.05 was considered significant.

## Results

All the participants underwent routine systemic and ophthalmological examinations. The physical characteristics of participants such as age, body mass index (BMI) and myopic diopter, were collected. OBF, HR and BP were measured before and after dobutamine injection (table 1). BMI was calculated as weight in kilograms divided by the square of the height in metres. The statistical results showed that HR, systolic blood pressure (SBP) and diastolic blood pressure (DBP) were significantly increased (all *p* values < 0.001).

**TABLE 1:** Summary of participant characteristics.

Characteristics	Data	*p*-value
No. of males/females	40/3	—
Age, years	26.03 ± 3.07	—
BMI, $kg/m^{2}$	22.58 ± 2.58	—
Myopic, diopter	−2.79±-2.04	—
HR before injection, bmp	68.33 ± 10.92	*p* < 0.001
HR after injection, bmp	84.92 ± 16.45	
SBP before injection, mmHg	119.54 ± 10.23	*p* < 0.001
SBP after injection, mmHg	158.95 ± 13.30	
DBP before injectio, mmHg	74.36 ± 8.17	*p* < 0.001
DBP after injection, mmHg	83.87 ± 8.11	

Data are reported as mean ± standard deviation unless otherwise specified.

**FIGURE 3:**
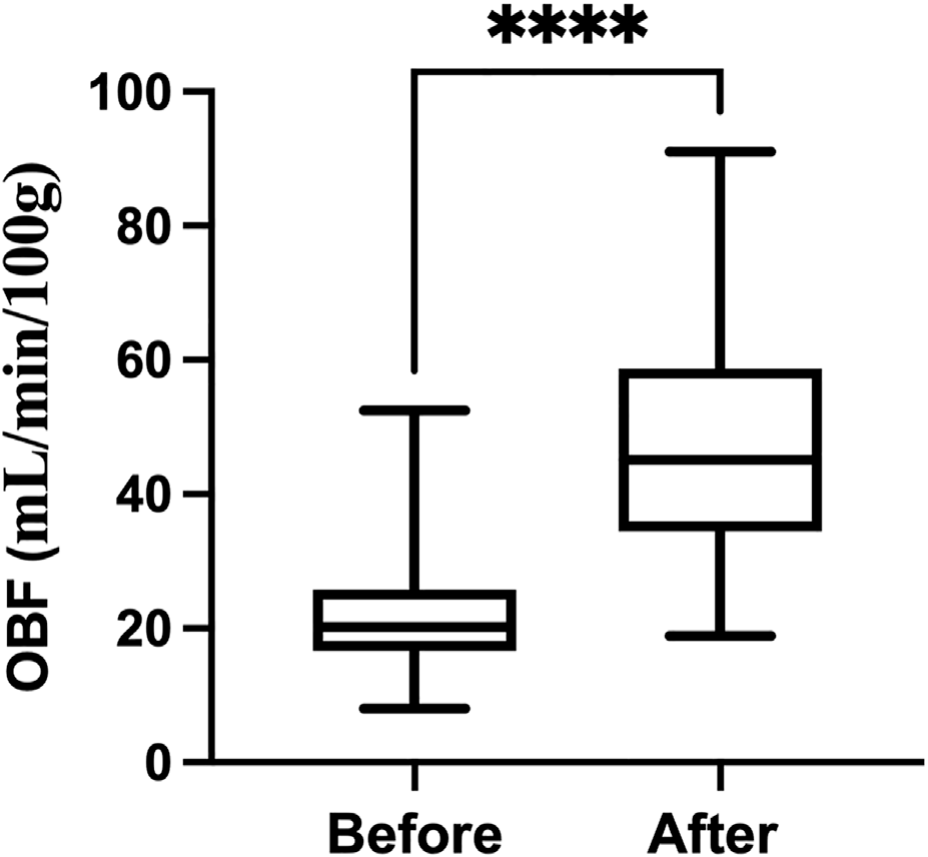
Changes in OBF before and after dobutamine injection.

Figure 3 shows the dobutamine induced a significant increase in OBF (from 22.43 ± 9.87 to 47.73 ± 14.02 ml/min/100g, *p* < 0.0001).

The differences of HR, SBP, and DBP between the period before and after dobutamine injection, and basic value (age, myopia, BMI) are as covariates into the logistic regression, with the difference in OBF as the dependent variable into the logistics regression model. Only one eye of each subject was randomly included. The results showed that age, HR and SBP were the main factors affecting the changes in OBF (Table 2).

**TABLE 2:** Results of logistics regression analysis.

Variable	β	SD	Wald	p	OR	95% CI
Age	0.434	0.196	4.879	0.027^a^	1.543	1.050–2.268
Myopic	0.002	0.002	0.683	0.409	1.002	0.997–1.007
BMI	0.362	0.188	3.692	0.055	1.436	0.994–2.098
HR	0.096	0.044	4.768	0.029^a^	1.100	1.010–1.199
SBP	0.121	0.059	4.201	0.040^a^	1.128	1.005–1.266
DBP	0.017	0.060	0.077	0.781	1.017	0.904–1.144

^a^ *p* < 0.05.

OR, odds ratio; SD, standard deviation; CI, confidence interval.

## Discussion

This study firstly explored the relationship between dobutamine and OBF in healthy participants using the 3D-pcASL. After intravenous dobutamine treatment OBF was significantly increased, and hemodynamic parameters (HR, SBP and DBP) were more than 10% higher than the baseline level. Several significant correlations resulted from the multivariate model, such as HR, SBP and age. We hypothesis dobutamine increases OBF by stimulating retinal vasodilator responses and causing changes in hemodynamic parameters.

From pharmacology aspect, dobutamine is a sympathomimetic amine, sympathetic and parasympathetic nerves have been identified in the choroid (Polska et al., 2007). Accordingly, the changes of OBF may depend on nervous stimulation, we hypothesize neural component is involved in the regulatory mechanism. Also, based on the widespread distribution of β -ARS in retinal vessels and neuroretinas, β -ARS is believed to play an important role in retinal vascular and neuronal functions (Ruan et al., 2020; Mori et al., 2017) provide the evidence that stimulation of β-adrenoceptors leads to dilation of small arteries in the rat retina, thereby increasing retinal blood flow. Dobutamine has the capacity to stimulate β-adrenoceptors in the cardiovascular system, may also to dilates retinal blood vessels of human.

From risk factor aspect, first, a higher dose of dobutamine leads to an increase in cardiac output, resulting in a faster heart rate in healthy subjects. One formula for OBF is OBF = HR×OPA×0.00149, where OPA indicates the maximum ocular pulse amplitude (Saha et al., 1993). It can be seen that heart rate is one of the important factors affecting OBF. The use of therapeutic agents, such as atropine, more attention should be paid to the ocular perfusion status while HR is increased for a short period of time. Furthermore, many large population-based studies showed a positive relationship between blood pressures, especially SBP, and intraocular pressure (IOP) (Bonomi, 2000; Xu et al., 2007). Changes in BP can lead to small changes in atrial fluid formation that may increase capillary pressure in the ciliary body (Bill 1973). In other words, choroid perfusion pressure adjustments induced by changing BP can produce better choroid blood flow compensation (Kiel, 2012). For example, high systemic blood pressure at the beginning of the glaucomatous process stimulates metabolism and positively contributes to OBF regulation and auto-regulation. In our study, changes in systolic BP were significantly correlated with changes in OBF. Therefore, we hypothesize that SBP signals drive IOP to adjust OBF. In addition, to adapt to the metabolic needs during changes in visual activity, OBF is highly regulated to compensate for different perfusion pressures (Wiącek et al.,Ravalico et al., 1996;^2020^) found that OBF decreased with age in normal people, possibly because systemic vascular resistance increased with age. Furthermore, we find age is a risk factor for automatic regulation of OBF, the vascular structural weakening effects of aging provide a mechanistic explanation.

Meanwhile, many researchers have consistently reported that people with myopia have lower blood flow parameters regardless of the diagnostic method (Grudzińska and Modrzejewska 2018). As the axial length of the eye increases, the thinning and loss of choroidal and retinal structures in myopic patients further reduce their oxygen demand, resulting in reduced choroidal blood flow (Zhao et al., 2022). In the present study, the degree of myopia of the subjects did not contribute significantly to the change in the magnitude of OBF after dobutamine injection, probably because the vast majority of subjects in the present study were not so myopic as to have abnormal perfusion. Besides, obesity has been reported to be an independent risk factor for high IOP (Ramya et al., 2018). However, an increase in BMI results in an nonsignificant increase in IOP in healthy subjects (Sedaghat et al., 2017). In other words, there is no highly association between normal BMI and OBF, which is in accordance with our findings.

Our study has some limitations. First, our study limited to healthy participants. Dobutamine may have different effects in patients with ocular diseases (e.g., glaucoma, anterior optic nerve ischaemic lesions), so further studies are needed to investigate the effects of dobutamine on OBF in different patient groups. Secondly, OBF is associated with sex, and sex hormones are thought to be involved in the regulation of OBF (Geyer et al., 2003). The sex differences would be more pronounced or of similar magnitude if the participants had an even male:female ratio. To further explore the effect of other risk factors on changes in OBF after dobutamine injection, more subjects with distinct individualized characteristics need to be enrolled. Finally, selection of PLD is central to ASL performance and interpretation, and relies on estimation of mean arterial transit time (ATT) from the labeling region to the tissue compartment (Bambach et al., 2022). OBF velocity may change in participants after dobutamine injection and shorten the ATT, we will try different PLD in our future works.

In conclusion, our data indicated that intravenous dobutamine significantly increased OBF that quantified by 3D-pcASL, a non-invasive method. Regarding risk factors of OBF when using dopamine homologues, age, HR and SBP should be considered. Dobutamine could become an option to increase OBF and counteract ocular vascular ischaemia, may open a new therapeutic avenues.

## Data Availability

The original contributions presented in the study are included in the article/supplementary material, further inquiries can be directed to the corresponding author.
